# Biobanking across Africa: from participant-level trust to institutional trustworthiness

**DOI:** 10.3389/fgene.2026.1953975

**Published:** 2026-10-16

**Authors:** Aisha Sallah, Salma Tarmoun, Krishna Gajjar, Patience Yeboah, Bernard Abakah, Everlyne Mutua, Dimitris Boufidis

**Affiliations:** 1 Penn Science Policy and Diplomacy Group, University of Pennsylvania, Philadelphia, PA, United States; 2 Department of Genetics, Perelman School of Medicine, University of Pennsylvania, Philadelphia, PA, United States; 3 Applied Mathematics and Computational Science, School of Arts and Sciences, University of Pennsylvania, Philadelphia, PA, United States; 4 Department of Neurology, Perelman School of Medicine, University of Pennsylvania, Philadelphia, PA, United States; 5 Department of Bioengineering, School of Engineering and Applied Sciences, University of Pennsylvania, Philadelphia, PA, United States; 6 Department of Chemistry, School of Arts and Sciences, University of Pennsylvania, Philadelphia, PA, United States; 7 Department of Biology, School of Arts and Sciences, University of Pennsylvania, Philadelphia, PA, United States

**Keywords:** Africa, biobank, data, disease, ethics, governance, public health, research

## Abstract

Building trust and maintaining participant engagement are widely recognized as requirements for sustainable biobanking. Yet efforts to achieve these goals must account for the historical, economic, geo-political and socio-cultural contexts that shape participant engagement. In Africa, biobanking has shown immense scientific and global public health value, but persistent governance challenges continue to affect participation. In this Perspective, we examine the factors that have constrained participant engagement in biobanking across Africa. We argue that trust is not simply a participant-level deficit to be corrected through increased outreach. Instead, trust and mistrust are shaped by the broader biobanking ecosystem, and by whether its institutions have demonstrated that they are worthy of public trust. Drawing on studies from across the continent, we propose strategies for how governance bodies, researchers and healthcare professionals can develop biobanking systems and frameworks that are sustainable and accountable to the public at both the national and international levels. Importantly, we identify science diplomacy as a valuable mechanism for strengthening cross-border coordination in support of more equitable, coherent and locally-governed biobanking initiatives.

## 1 Introduction

Biobanks are organized repositories that link biological materials to relevant health information, such as clinical records, lifestyle, and genomic data (Malsagova et al., 2020). They play a vital role in advancing biomedical research by enabling large-scale studies on health and disease. Prominent examples of biobanking successes include the UK biobank (Sudlow et al., 2015), Biobank Japan (Nagai et al., 2017), and the All of Us Program in the United States (All of Us Research Program Investigators, 2019). Similar efforts continue to expand across the African continent (Nanyonga et al., 2026). The H3Africa Consortium (2012–2022), for instance, supported 51 projects spanning 30 countries (Sengupta et al., 2025). The initiative published over 700 peer-reviewed articles, unveiled 3 million genetic variants (Choudhury et al., 2020), established disease surveillance hubs (Ekoru et al., 2016), and fostered a generation of African scientists (Dandara et al., 2014).

Despite H3Africa’s substantial impact, the position of the continent in global biobanking remains weak, with African populations representing less than 10% of global biomedical databases (Winter et al., 2026). This lack of representation is rooted in the regulatory, ethical, infrastructural, technological, and financial obstacles that biobanks currently face (Staunton and Moodley, 2013). A critical and frequently identified issue is the lack of public trust in the broader biobanking infrastructure (Moodley and Singh, 2016; Amoakoh-Coleman et al., 2023). Throughout this paper, we refer to trust as participants’ confidence that their biological samples will be handled responsibly, only for agreed purposes, that institutions will act in good faith and that the outcomes will meaningfully benefit them, their communities, or public health more broadly. Yet with a historical pattern of biomedical extractivism, in which biological material and data flow outward often without local benefit (Staunton and de Vries, 2020), the conditions for such confidence have not always been met.

Bioethicists and researchers have argued that robust communication, outreach, and ethical governance are key to strengthening public trust in biobanking (Moodley and Singh, 2016; Amoakoh-Coleman et al., 2023). In this Perspective, we revisit the notion of trust and emphasize that these measures, while necessary, are insufficient in the context of biobanking in Africa. In Section I we describe this context, shaped by historical and socio-cultural factors. In Section II, rather than asking primarily how communities can be persuaded to trust biobanking, we ask what key actors must do to become worthy of that trust. We then translate this principle into actor-specific responsibilities and operational frameworks that prioritize accountability at both domestic and transnational levels.

## 2 Section I: beyond trust–factors shaping engagement with biobanking in Africa

### 2.1 Information

The information gap is a fundamental and frequently highlighted barrier to participation. Individuals who lack knowledge about biobanking and scientific research cannot make an informed decision regarding their participation. This does not necessarily reflect a lack of trust, but it is critical that this gap be addressed before trust can be established. Shortcomings of the informed consent process may leave participants uncertain about what participation involves and how it may affect them depending on their socioeconomic circumstances, health status, or other factors. Questions might remain about how samples and data are processed, stored, maintained, and shared; what information may be generated from them; why they are scientifically valuable; and why samples must be retained after the initial analysis. Perceived understanding routinely outruns objectively measured understanding, and the least-understood elements are often the load-bearing ones: risks, confidentiality, and compensation (Amayoa et al., 2022). A study of participant understanding in a community-based health screening and biobank platform in South Africa illustrates this, where participants who report understanding a study may recall nothing of its biobanking components, or may have simply enrolled for screening and reimbursement rather than the research use they are consenting to (Ngwenya et al., 2020). This is not a gap in literacy—comprehension tracks with how information is conveyed, in what language, and over how much time. Importantly, this challenge is not unique to Africa, as limited consent comprehension has also been associated with reduced participation in high-income settings (Prictor et al., 2018; Davis et al., 2019; Ahn et al., 2025; Lee et al., 2025).

### 2.2 Culture and religion

Engagement with biobanks is also shaped by cultural and religious beliefs about blood, tissue, and what happens to a sample once it leaves the body (Amoakoh-Coleman et al., 2023). The heterogeneity in beliefs regarding biobanks across communities illustrates the complexity of cultural barriers to participation. In a study by Barchi et al. (2015), for example, some participants described sample donation as an act of social responsibility, while others compared donating biological material to male circumcision, where removed tissue was believed to circulate and be misused by others (Amoakoh-Coleman et al., 2023). A Cameroonian ethnographic study similarly identified the fear of ritual use of donated blood as a deterrent to participation, alongside needle pain, religious beliefs, and limited transparency from health personnel (Asahngwa et al., 2025). These concerns should not be treated simply as misinformation, as they may be closely connected to local understandings of health and disease. Consent processes may further compound these concerns when biobanking and genomic research are not explained in a culturally sensitive manner. A 2025 systematic review of informed consent in genomic studies across sub-Saharan Africa found that comprehension challenges were shaped not only by health and research literacy but by community decision-making norms (Li et al., 2025). In some communities, an emphasis on individual autonomy sits uneasily with more communitarian approaches, meaning that informed consent should be supported by comprehensive and authentic community engagement (H3Africa Consortium, 2017).

### 2.3 History of colonization and exploitation

Any account of mistrust that stops at myth or illiteracy is incomplete. Distrust of Western-linked medical research on the continent has a history that predates contemporary biobanking. Colonial medical campaigns frequently subjected communities to mandatory examination and treatment, sometimes under threat of violence. Historical exposure to these campaigns has been linked to lower vaccination rates and weaker health-intervention uptake generations later (Lowes and Montero, 2021). African biomedical research took shape inside colonial systems in which priorities were set by European administrations, accountability to the participants was weak, and coercion was routine, with consent rarely sought (Tilley, 2016). These asymmetries persisted after independence, as much health-research funding continued to originate from Global North partners, often giving them disproportionate control over research agendas, samples, and authorship (Matenga et al., 2019). This is often referred to as “helicopter research”, where samples and data travel outward while source institutions and communities retain limited authority and benefit (Nguah and Lartey, 2024).

This dynamic recurs across research settings. The 2009 Kano litigation alleged that consent for Pfizer’s trovafloxacin study became procedural rather than substantive (Abdullahi v. Pfizer, Inc., 2009). The publication of four San genomes in 2010 sparked community objections over verbal consent, pejorative descriptions, and cultural disclosures irrelevant to the findings (Schuster et al., 2010; Chennells and Steenkamp, 2018). The 2019 Wellcome Sanger dispute, in turn, exposed ambiguity over ownership, commercialization, and future uses (Galasso and Geiger, 2025). More recently, 54gene’s 2023 collapse and litigation surrounding the control and transfer of its genomic holdings revealed the stewardship risks when a commercial biobank fails (Walker, 2026). These are not merely isolated incidents confined to the past. Empirical evidence shows that these concerns continue to affect biomedical research on the continent today. Ugandan researchers report that cross-border sharing can impede local capacity and occur under unclear governance and inequitable benefit-sharing, while African pathogen-genomics stakeholders describe informal rather than enforceable arrangements (Kaawa-Mafigiri et al., 2023; Selormey et al., 2025). Kenyan and Ugandan studies likewise identify uncertainty about benefit sharing, data access, ownership, and intellectual-property rights (Sudoi, 2024; Sebatta et al., 2026).

Taken together, these cases reveal a structure in which priorities, decision-making authority, credit, and benefits flow outward while risks and limited control are left with participating communities. Mistrust, in this context, is therefore not a psychological or cultural barrier but a rational assessment of whether institutions have demonstrated that they will respect agreed limits, share benefits, prevent unauthorized uses, and provide remedies when obligations are breached. The response then, is not to persuade communities to trust research more, but to address the conditions that make such distrust warranted.

## 3 Section II: strategies to strengthen accountability and roles of key actors

### 3.1 From relational trust to institutional accountability

When analyzing hesitancy towards engagement in biobank-related projects, it is critical to examine the entities that people are being asked to trust and how far that trust may extend. In practice, participants encounter professionals who work with them directly: a nurse who has cared for them, a familiar clinician or a community liaison. Therefore, participant trust rests on relational trust that is developed through direct familiarity, care, and/or shared community ties (Figure 1).

**FIGURE 1:**
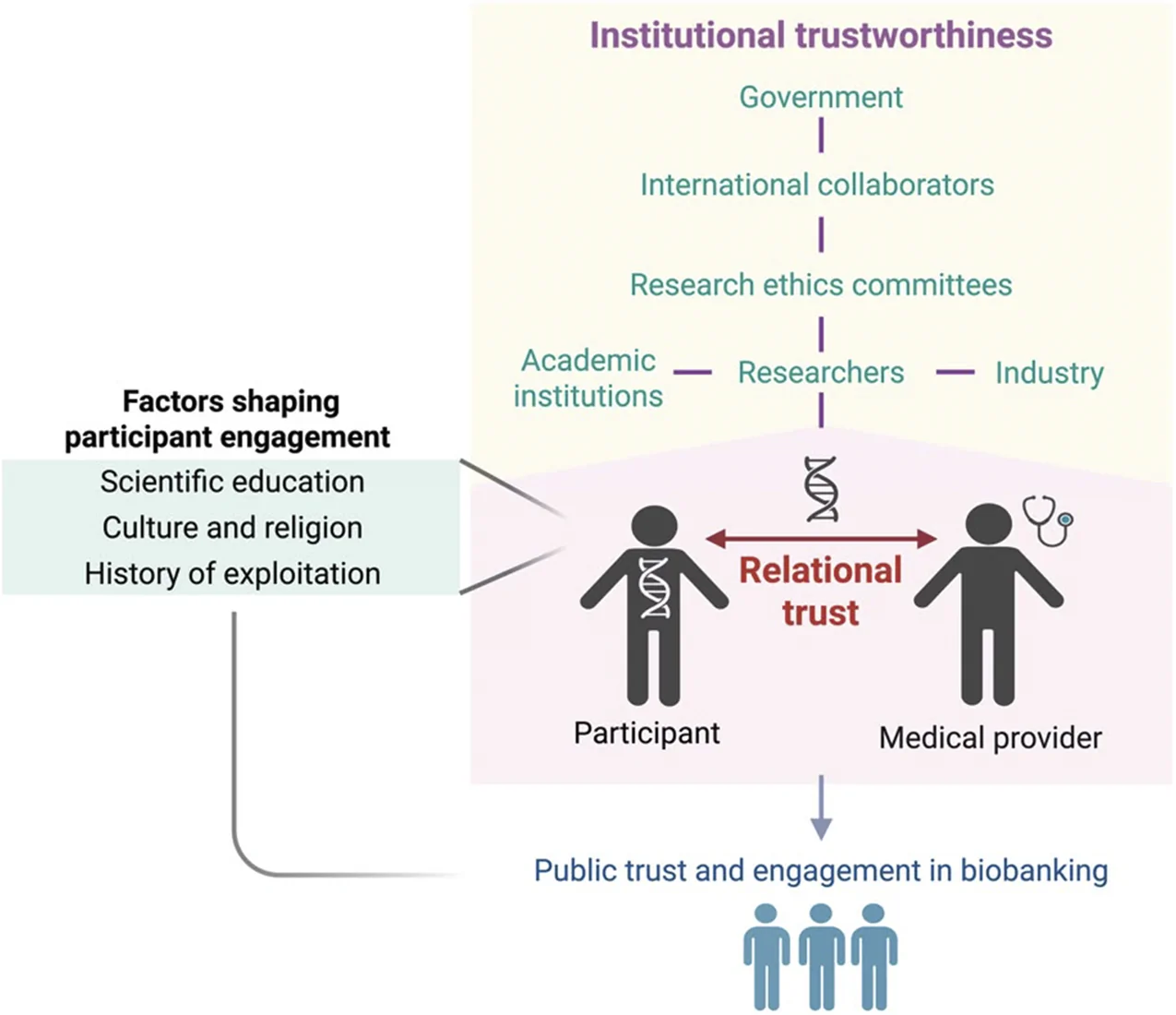
The biobanking ecosystem: Institutional trustworthiness differs from relational trust. While trust generally refers to participants’ confidence in the broader biobanking system, in practice this confidence is often shaped more directly by interactions with medical providers or researchers, alongside socio-cultural and historical factors. This relational trust, while meaningful, does not automatically extend to the institutions operating beyond that relationship, which must independently demonstrate trustworthiness and accountability to participants.

Biobanking, however, requires trust in a largely invisible chain: the principal investigator, sponsoring institution, research ethics committee, data analysis team, international collaborators, commercial partners, regulatory bodies, as well as secondary researchers that arrive years later. Participant trust then, built on close relationships and direct interactions, cannot be considered to extend across this entire biobanking chain. Once samples and data progress beyond the relationship in which participation was negotiated, trustworthiness, i.e., consistently maintaining the conditions for trust, must be secured through governance.

The following section evaluates the responsibilities of the principal stakeholders across the biobanking chain, presenting strategies for how each entity should operate such that participant trust is maximized and all scientific insights gained from biobanking positively impact participating communities.

### 3.2 Government

The gaps identified in the previous subsection can only be sealed by a public authority. A consortium framework binds only its members; a grant-funded committee lasts as long as the grant. A national regulator has neither limit: it persists across projects and acts with the standing authority of a state. Governments therefore have a core duty to protect participants. This requires adequate resources, operational independence and authority to license and track biobanks, audit storage and data-security practices, investigate complaints, impose sanctions that carry consequences, review and approve international transfers before samples or data leave the country. At minimum, every licensed biobank and approved transfer should be publicly recorded and traceable. Trustworthiness should also be developed at both domestic and transnational levels. As samples and data often extend beyond the reach of any single national regulator, governments have a distinct coordinating role across jurisdictions.

Dedicated scientific expertise within government agencies is necessary, though existing African models demonstrate that this capacity need not take a uniform form: Rwanda incorporates science diplomacy into its national Science, Technology and Innovation policy (National Council for Science and Technology, 2020), South Africa has established dedicated Science and Technology representation within its diplomatic service (Department of International Relations and Cooperation, 2026), and the African Union has recommended regional STEM diplomacy offices and pooled scientific expertise (African Union Commission and UNESCO, 2024). Countries can scale science diplomacy capacity to available resources by assigning science and technology responsibilities to existing officials, sharing specialist expertise regionally, or deploying dedicated science counselors in strategically important missions. Such science diplomacy mechanisms can complement national regulators by facilitating coordination among relevant authorities when scientific collaborations, samples, and data span jurisdictions.

### 3.3 Research ethics committees

Research ethics committees (RECs) are regulatory groups that monitor biobanking projects, comprising scientists, medical providers, lawyers, and community representatives (Mehta et al., 2023). Their primary objective is to protect the rights, privacy, and safety of participants, thereby playing a central role in establishing public trust.

A key function of RECs is to establish guidelines for informed consent. As emphasized previously, consent must be obtained in a culturally sensitive and comprehensible manner. Additionally, RECs must verify that consent acquisition is performed systematically. In the African context specifically, ethicists have argued for the implementation of a tiered consent model, in which participants can choose to donate their samples for single-time use or for wider use in future studies, with or without re-consent (Nembaware et al., 2019). This tiered approach equips individuals with a greater sense of autonomy over their samples. If they lose trust in the research or have concerns regarding the utility of their samples, they can choose to withdraw and not re-enroll. Given that consent is a pillar of trust, RECs must establish consent guidelines meticulously and remain transparent at every stage of the biobanking process.

Beyond consent, RECs are responsible for developing frameworks to regulate confidentiality as well as the cross-border export of samples (Staunton and Moodley, 2013). RECs must also retain records of reviews and carve out Standardized Operating Procedures, ensuring that all associated stakeholders follow set rules and that processes are streamlined. With concerns of “helicopter” research in Africa, RECs must also establish formal reporting mechanisms and strictly address any complaints (Singh and Moodley, 2021). If any ethical issues arise, RECs must act immediately to halt research, review facts, and inform participants. The establishment of a rigorous, competent REC is central to cultivating trust, as the quality of ethical review shapes the course of a study and eventual trust outcomes.

### 3.4 Scientific researchers and medical providers

Physicians, scientists, and other medical professionals hold a high degree of public trust, playing a significant role in shaping how individuals understand research opportunities (Pickering et al., 2026). Patients may first encounter research through clinical visits and may be more willing to consider participation when a study is introduced by a trusted provider. However, this overlap between clinical care and research may become an ethical challenge, especially if the individual collecting a sample is or is perceived to be responsible for the participant’s care. This can lead to a misconception, in which participants mistakenly believe that research is conducted for their immediate clinical benefit (Appelbaum et al., 1987). For example, in the KwaZulu-Natal platform, several participants believed they were enrolling for a free health screening rather than for research purposes (Ngwenya et al., 2020). Providers therefore have a responsibility to clarify the distinction between clinical care and research (Kombe et al., 2016).

Beyond this, an important priority is sustained investment in the capacity of frontline providers to act as informed ethical agents rather than trusted recruitment conduits. This demands that healthcare professionals are adequately informed about the purpose, risks, benefits, and broader implications of the study in question. Scientists therefore carry substantial informational responsibility, as both producers and disseminators of knowledge. Researchers must ensure that healthcare providers understand not only the immediate aims of a study but also its public health significance. Stronger collaboration between researchers and medical professionals is therefore critical. Together, they can empower participants by closing the information gap and supporting autonomous decision-making through accurate, transparent, and ethically grounded communication. This would represent a meaningful shift in how public engagement with biobanking is approached.

This shift can further be reinforced through broader investments, including expanding scientific education and training in schools and universities and developing evidence-informed policies that demonstrate the role of science in public decision-making, particularly during health crises. Creating incentives that enable early-career researchers, particularly those in the diaspora, to contribute expertise, develop sustained international collaborations or even return to establish locally anchored research programs can also help strengthen institutional connections, facilitate knowledge exchange, and support science-diplomacy initiatives.

## 4 Discussion: aligning interests and benefits

Each of the aforementioned actors possesses diverging interests. Researchers may be motivated by publications and grant renewals, while institutions prioritize data acquisition and intellectual property. Additionally, commercial entities are primarily driven by profit, governments may not always prioritize scientific advancement in the form of biobanking, and communities seek protection, respect, and tangible benefits. Public trust lacks a single actor with sufficient motivation to protect it. A study from Uganda showed how these interests diverge in North-South collaborations, highlighting power asymmetries, weak bargaining positions for local institutions, and researchers’ concerns about losing control of samples once they cross borders (Mwaka et al., 2022). Strategies to align these interests thus must be prioritized in order to reach the collective goal of “trustworthy” biobanking. The first strategy is to establish benefit-sharing as an explicit, binding condition from the outset. These arrangements, whether in the form of capacity-building, infrastructure development, or a defined share in downstream commercial value, should be negotiated prior to the collection of samples and incorporated into strict material transfer agreements. The source community must be recognized as a party to these agreements, not as an afterthought (Mwaka et al., 2022; Bedeker et al., 2022). This approach links each actor’s rewards to the community’s benefits, ensuring that the success of researchers or partners is directly connected to positive outcomes for the community.

A second strategy is to establish equal partnership as the foundational norm for collaboration. Ethical research benchmarks emphasize partnerships characterized by mutual respect, reduced disparities in capacity and status, meaningful local involvement in setting research agendas, and fair benefit distribution (Emanuel et al., 2004). In practice, this requires local co-leadership, authorship, and joint decision-making, rather than restricting local institutions to sample provision. These arrangements provide African institutions with a substantive stake in research outcomes. Diaspora scientists may support these partnerships by connecting institutions across countries and helping navigate differences in scientific, regulatory, and funding systems. Their cross-contextual knowledge and institutional networks can be valuable in this facilitative role (Echeverría-King et al., 2022).

Community-led efforts to establish ethical governance frameworks within these partnerships are already present on the continent. The San Code of Research Ethics stands as a notable example, establishing community-defined principles and expectations for researchers working with San communities (Schroeder et al., 2019). African RECs have similarly played a crucial role in developing contextually grounded approaches to informed consent, community engagement, ethics oversight, and biospecimen and data governance within the H3Africa Consortium (de Vries et al., 2015; Yakubu et al., 2018; Tindana et al., 2019). Beyond collaborations, African-led efforts to harmonize governance, ethical frameworks, and policies are growing, as demonstrated by PABNet’s work to strengthen biobanking expertise, quality systems, professional networks, and governance capacity across the continent (Nanyonga et al., 2026).

A third approach is to ensure that trustworthiness becomes an advantageous course of action. The proposed public national registry and transparency infrastructure support this by making ethical conduct visible and breaches traceable. Consequently, reputational incentives begin to reward actors who adhere to ethical standards, and access to funding and authorization can be made contingent upon demonstrated benefit-sharing and ongoing auditability. This approach must be complemented by awareness-building initiatives, as the concept of benefit-sharing remains poorly understood among many regional stakeholders and cannot serve as a unifying principle unless it is first recognized (Munung and de Vries, 2020).

## 5 Conclusion

Biobanking serves as a cornerstone for biomedical research and Africa’s high genetic diversity and substantial disease burden uniquely position the continent to drive discoveries of global impact. Here, we briefly described the state of biobanking across the African continent, addressing a key challenge that constrains participation: a lack of trust. This challenge arises from the lack of accessible information, heterogeneous cultural beliefs, a history of exploitation, as well as the lack of enforcement strategies to protect the rights, privacy, and safety of participants. To cultivate trust, we emphasize that every stakeholder in the process, from governments and RECs to scientists and medical providers, must possess a shared sense of accountability and work collaboratively to align interests such that biobanking is performed ethically. Ultimately, these efforts can strengthen participant engagement, support sustainable research governance across Africa, and foster shared-benefit partnerships to increase African representation in biomedical datasets.

## Data Availability

The original contributions presented in the study are included in the article/supplementary material, further inquiries can be directed to the corresponding authors.
